# Macroepidemiological aspects of porcine reproductive and respiratory syndrome virus detection by major United States veterinary diagnostic laboratories over time, age group, and specimen

**DOI:** 10.1371/journal.pone.0223544

**Published:** 2019-10-16

**Authors:** Giovani Trevisan, Leticia C. M. Linhares, Bret Crim, Poonam Dubey, Kent J. Schwartz, Eric R. Burrough, Rodger G. Main, Paul Sundberg, Mary Thurn, Paulo T. F. Lages, Cesar A. Corzo, Jerry Torrison, Jamie Henningson, Eric Herrman, Gregg A. Hanzlicek, Ram Raghavan, Douglas Marthaler, Jon Greseth, Travis Clement, Jane Christopher-Hennings, Daniel C. L. Linhares

**Affiliations:** 1 Veterinary Diagnostic and Production Animal Medicine, Iowa State University, Ames, Iowa, United States of America; 2 Swine Health Information Center, Ames, Iowa, United States of America; 3 Veterinary Population Medicine, University of Minnesota, Saint Paul, Minnesota, United States of America; 4 College of Veterinary Medicine, Kansas State University, Manhattan, Kansas, United States of America; 5 Veterinary & Biomedical Sciences Department, South Dakota State University, Brookings, South Dakota, United States of America; Columbia University, UNITED STATES

## Abstract

This project investigates the macroepidemiological aspects of porcine reproductive and respiratory syndrome virus (PRRSV) RNA detection by veterinary diagnostic laboratories (VDLs) for the period 2007 through 2018. Standardized submission data and PRRSV real-time reverse-transcriptase polymerase chain reaction (RT-qPCR) test results from porcine samples were retrieved from four VDLs representing 95% of all swine samples tested in NAHLN laboratories in the US. Anonymized data were retrieved and organized at the case level using SAS (SAS^®^ Version 9.4, SAS^®^ Institute, Inc., Cary, NC) with the use of PROC DATA, PROC MERGE, and PROC SQL scripts. The final aggregated and anonymized dataset comprised of 547,873 unique cases was uploaded to Power Business Intelligence—Power BI^®^ (Microsoft Corporation, Redmond, Washington) to construct dynamic charts. The number of cases tested for PRRSV doubled from 2010 to 2018, with that increase mainly driven by samples typically used for monitoring purposes rather than diagnosis of disease. Apparent seasonal trends for the frequency of PRRSV detection were consistently observed with a higher percentage of positive cases occurring during fall or winter months and lower during summer months, perhaps due to increased testing associated with well-known seasonal occurrence of swine respiratory disease. PRRSV type 2, also known as North American genotype, accounted for 94.76% of all positive cases and was distributed across the US. PRRSV type 1, also known as European genotype, was geographically restricted and accounted for 2.15% of all positive cases. Co-detection of both strains accounted for 3.09% of the positive cases. Both oral fluid and processing fluid samples, had a rapid increase in the number of submissions soon after they were described in 2008 and 2017, respectively, suggesting rapid adoption of these specimens by the US swine industry for PRRSV monitoring in swine populations. As part of this project, a bio-informatics tool defined as Swine Disease Reporting System (SDRS) was developed. This tool has real-time capability to inform the US swine industry on the macroepidemiological aspects of PRRSV detection, and is easily adaptable for other analytes relevant to the swine industry.

## Introduction

The most economically important swine disease in the United States (US) is porcine reproductive and respiratory syndrome (PRRS), causing annually losses of more than 600 million dollars[1]. PRRS was first reported in the US in the late 1980s as mystery swine disease [2]. In the early 1990’s, the etiology of the disease was confirmed as PRRS virus (PRRSV) [3] in Europe [4, 5] and reproduction of the disease in gnotobiotic pigs was performed shortly after in the US [6]. In the 30 years since the introduction of PRRSV to the US swine herds, the virus has become widespread in the US and is present in most of the major pork producing countries.

Producers, veterinarians, and researchers are highly motivated to better understand the occurrence, detection, and geographical distribution of PRRSV so that targeted disease control practices can be implemented. In response to that need, Dr. Robert Morrison developed the Swine Health Monitoring Project (MSHMP), which has been reporting PRRS weekly incidence and yearly cumulative incidence [7] and prevalence over time [8] of PRRSV from participating US swine breeding herds since 2009. As of December 2018, the MSHMP had 971 breeding herds enrolled, representing 2,824,933 sows [9]. The MSHMP project has provided useful information; however, it reports incidence data based on veterinarian’s judgement and is not necessarily confirmed by appropriate diagnostic testing. It also does not include information of more than 3.5 million breeding females from an inventory of 6.33 million breeding females, in the US not participating in the voluntary survey, and does not track pathogen detection in 68.2 million heads of market hogs, which represent 91.5% of the swine population in the US based on the December of 2018 USDA Quarterly Hogs and Pigs report [10]. Thus, there is still the need to further develop disease reporting systems to complement existing tools, better documenting the macroepidemiological aspects of PRRSV activity in the US swine population. Macroepidemiology has been described as the study of multiple inputs of national disease pattern and determinants [11, 12] Similarly, for the purpose of this work the macroepidemiological term is used to describe the characteristics associated with the detection of PRRSV RNA by real-time reverse-transcriptase polymerase chain reaction (RT-qPCR) at major veterinary diagnostic laboratories (VDLs). Characteristics include period of time, geographical region (US state), age group, and specimen.

Multiple VDLs in the US have independently, inconsistently and intermittently reported data on PRRSV RNA detection in samples, usually summarized in different formats including peer-reviewed publications, professional organization presentations and proceedings, lay-journal communications, and VDL summaries. To the best of our knowledge, there is no single source of information currently available in the US where standardized information regarding sample type, geographical region, age category, diagnostic test information, and test results from different VDLs are gathered, aggregated, and reported in a consistent, routine and timely fashion. Therefore, the objective of this study was to document macroepidemiological aspects of PRRSV RNA detection by VDLs providing the major US swine diagnostic testing, using a standardized approach that can be routinely updated over time and provide summaries to the swine community. More specifically, standardized diagnostic and sample data were collected by participating VDLs, anonymized and aggregated to report patterns of PRRSV RNA detection over time, geographic regions, pig age groups, and sample types.

## Materials and methods

### Study design

**Fig 1:**
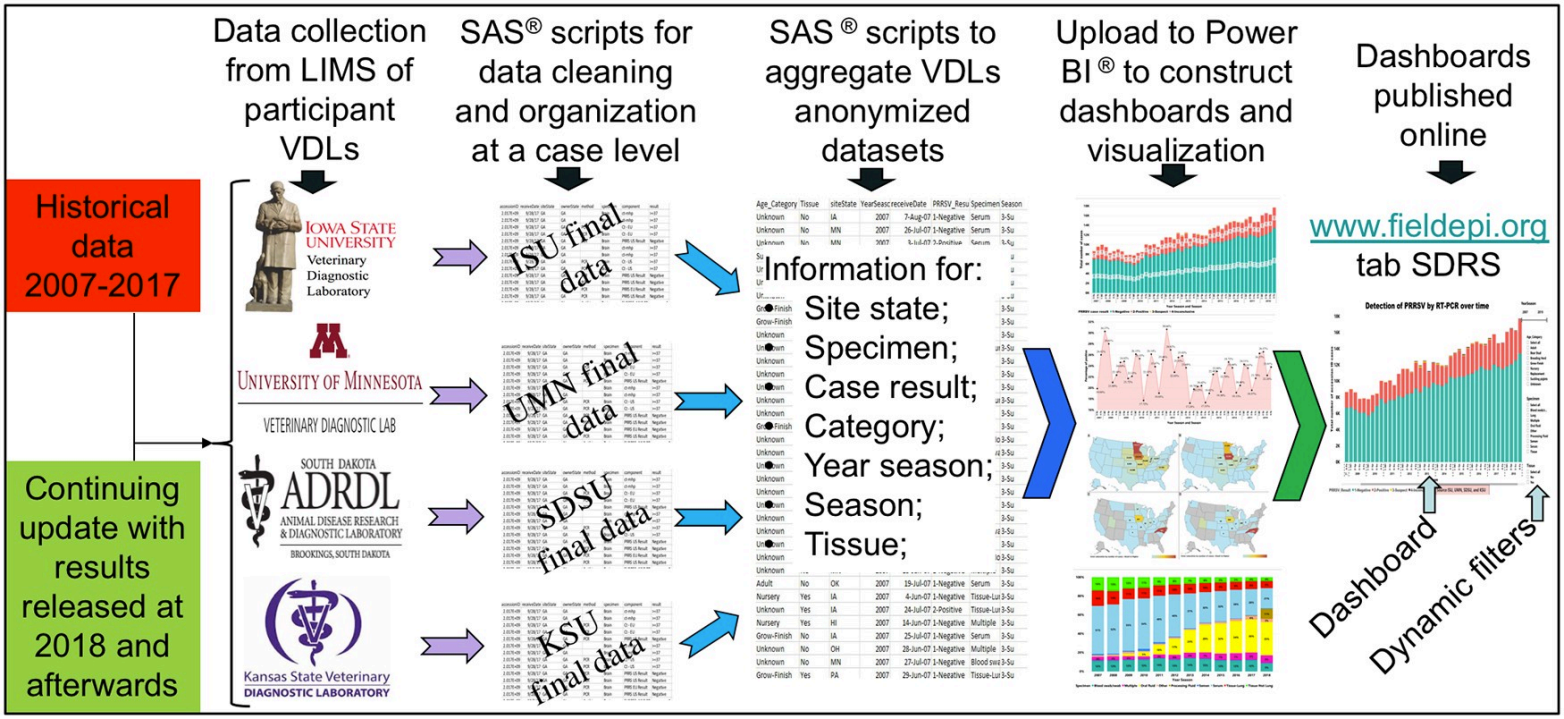
Swine Disease Reporting System schematic flow chart for data processing.

This was an epidemiological study of reporting patterns of PRRSV RNA detection by molecular techniques over time, geographic region, sample type, and age group in the US swine populations from June 2007 to November 2018. This was completed by collecting, aggregating, summarizing, and reporting standardized submission information and test results on PRRSV RNA detection by molecular diagnostic methods from participating laboratories including Iowa State University Veterinary Diagnostic Laboratory (ISU-VDL), University of Minnesota Veterinary Diagnostic Laboratory (UMN-VDL), Kansas State University Veterinary Diagnostic Laboratory (KSU-VDL), and South Dakota State University Animal Disease Research & Diagnostic Laboratory (SDSU-ADRDL). At the end of the data processing step an aggregated dataset was formed by the merging of data received from each of the VDLs into a consolidated dataset. Importantly, these participating VDLs account for >95% of porcine samples tested in National Animal Health Laboratory Network (NAHLN) VDLs in the US. Fields with information on producers, animal owners, veterinarians, farm premise identification number, and county location were not shared by the VDLs for confidentiality concerns. Data manipulation was done using SAS scripts (SAS^®^ Version 9.4, SAS^®^ Institute, Inc., Cary, NC) which allows updating the database consistently over time as part of the Swine Disease Reporting System (SDRS). The pathway and approach used to gather the data and to structure results are summarized below in the following sections: a) data source; b) definitions; c) receiving and processing data; and d) constructing dashboards, visualization charts, and online applications (apps) (Fig 1).

### Dataset source

Historical information from submission forms, test information, and test results for PRRSV RNA testing by molecular diagnostics were gathered and retrieved directly from the Laboratory Information Management System (LIMS) of each participating VDL. Retrieved data was shared using a Comma Delimited Value (CSV) or Health Level Seven International (HL7) structure. Historical data from 2007 to 2017 was retrieved from participating VDLs and shared by email on a CSV format. For 2018, CSV files containing updated results were retrieved from LIMS and shared by email on a monthly basis. Specifically, for ISU-VDL and UMN-VDL, in September of 2018 the HL7 messaging system was implemented instead of CSV files.

#### Definitions

**Fig 2:**
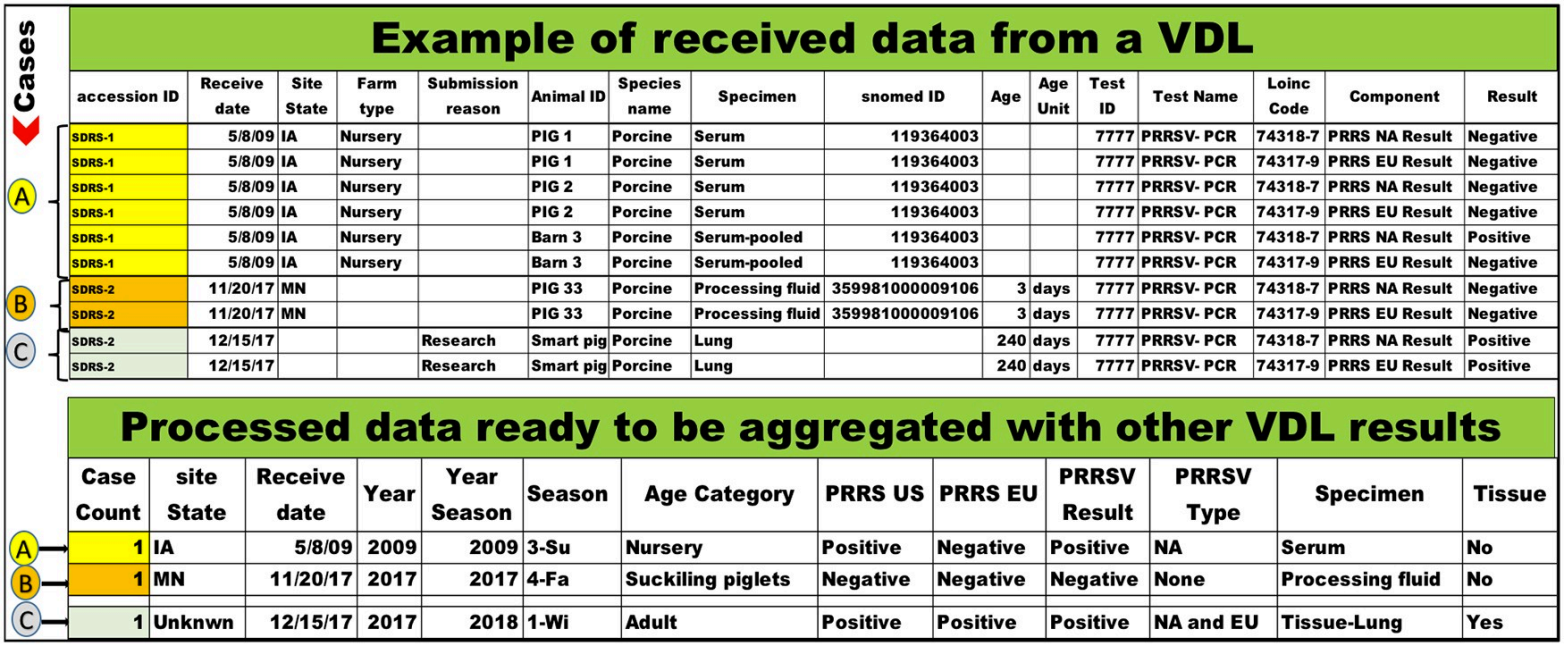
Schematic organization of raw data and final processed and anonymized information. Bullets with letters on the right represent a case.

For the purpose of this project the following definitions were applied:

1. Accession Identification Number (accession ID): corresponds to a unique set of characters assigned by each VDL to unambiguously identify samples during the submission process, testing procedures, and results communication (Fig 2). Each VDL has its own procedure to define the alphanumeric identifier used to create the accession ID. An accession ID may include a single sample, or multiple samples;
2. Case: for the purpose of this work, a case corresponds to each event of sample submission identified and related to a unique accession ID assigned by each VDL (Fig 2). All the results linked with one accession ID, represented by one or multiple samples submitted for diagnostic testing, were used to generate the final case result;
3. Site State: a state within the US where the pigs were physically located when the samples were collected for submission to a participating VDL (Fig 2);
4. Received date: corresponds to the day that the samples were received at the VDL (Fig 2);
5. Specimen: sample type submitted for testing. Examples include: oral fluid, processing fluid, serum, and lung (Fig 2);
6. Snomed CT: SNOMED CT is an international logic-based reference terminology used to present clinically relevant information. The SNOMED CT clinical terminology enables clinicians to record data with great accuracy and consistency[13]. SNOMED CT was used in this project as a way to standardize the identification of specimens across different VDLs. As an example, processing fluids (PF), the serosanguinous fluid recovered from piglet castration and tail docking [14] could be identified by text strings as Processing Fluid; Processing Fluid (pooling); Fluid, Testicle/Tail; Processing Fluids; Pool-Processing Fluids. With the use of SNOMED CT it has a standardized universal numerical identifier 359981000009106 (Fig 2);
7. Loinc Code: Logical Observation Identifiers Names and Codes (LOINC) is a universal standard code used for identifying test results (measurements) [15]. Each participant VDL mapped their local LIMS database’s test information to LOINC codes as a way to standardize the diagnostic data and enable for exchange, pooling, and processing of the diagnostic data (Fig 2);
8. PRRSV type: The PRRSV RT-qPCR test offers results for PRRSV type 1, also known as European PRRSV type (PRRS EU), and for PRRSV type 2, also known as North American PRRSV Type (PRRS NA). Results were tracked by LOINC codes, and test result string. Cases with RT-qPCR positive results for both PRRSV type 1 and 2 were defined as EU and NA. When the PRRSV type classification was not possible to be captured, the term not informed was applied (Fig 2);
9. Multiple: applied when more than one different term was described for the same variable in the same case (e.g. multiple specimens, or multiple age categories);
10. Unknown: information that could not be retrieved from the submission form, not included in the submission form by the submitter, or was not captured by the VDLs LIMS. Examples of fields with unknown results include site state, specimen, age category, PCR result for PRRSV type 1 and/or type 2 (Fig 2);
11. Advisory Council (AC): a group of swine veterinarians or producers from different swine production systems and geographical regions within the US, who volunteered time to evaluate the final information and give practical input and insights on interpretation of the major findings. Input from the AC was asked on a monthly basis during the development of this project in 2018.

#### Definitions for new variables created

1. Tissue: a data field with binary answer (yes or no) to indicate when the case contained at least one specimen that required pig euthanasia or biopsy for the collection (e.g. lungs, or lymph nodes). Considering that lung was the most common tissue submitted for PRRSV testing, the field tissue was further classified into Tissue-Lung or Tissue-Not Lung, allowing investigation on cases having only lung (Fig 2);
2. Cases result: A combination of LOINC code (identifies the test) and the test results was used to define the final case result. When LOINC codes were not present (some historical data), a combination of test name and test results was used to obtain the same information. When at least one sample within a case had a positive RT-qPCR result, the case was considered as positive. When there were no positive samples, but at least one sample resulting ‘suspect’, the test result from that case was classified as ‘suspect’. Similarly, when there were no samples testing positive or suspect and there was at least one sample testing ‘inconclusive’, the case result was classified as ‘inconclusive’. Finally, when there was no sample in the case testing positive, suspect, or inconclusive, and at least one sample was reported as ‘negative’, the case test result was reported as ‘negative’ (Fig 2). Different VDLs can potentially use a different cycle threshold (Ct) value to define a PCR results. For the purpose of this project, the final result was the one interpreted and reported by each VDL, as we considered that those assays were independently validated at each VDL. Thus, to ensure consistency, Ct values were not considered and not incorporated in the algorithm to classify case results;
3. Age Category: Age categories were defined by a two-step process. In step 1, information contained in the field ‘farm type’ was collected and generated eight categories defined as suckling piglets, nursery, grow-finish, breeding herd, boar stud, replacement, adult, or unknown. For cases where ‘farm type’ information was missing, the second step took place, where the ‘age’ data field was used to classify the age categories. Cases with age less or equal to 22 days old were classified as suckling piglets; 23 to 63 days as nursery; 64 to 200 as grow-finish; and above 200 days as adult. Cases for which the specimen was processing fluid were classified as suckling piglets. Cases for which none of the previous information were present were assigned age category as unknown (Fig 2).
4. Specimen: the primarily information used to define the specimen (aka sample type) was the snomed CT. For historical data where snomed CT information was not present, the field ‘specimen’ was used to report the specimen (Fig 2).
5. Season: season refer to winter, spring, summer, and fall months of the year. The results from December, January, and February were aggregated as winter, the results from March, April and May were aggregated as spring, the results from June, July and August were aggregated as summer, and the results from September, October and November were aggregated as fall. Received date was the basis to classifying a case according to a season and year. For example, results from December of 2017 were aggregated with the Winter Season of 2018 along with January and February of 2018, i.e., the winter season of 2018 started on December 1^st^ of 2017 and ended on February 28^th^ of 2018 (Fig 2).
6. Year season: A year season corresponds to a cycle of the four seasons starting in December and finishing in November of the upcoming year. Received date was the basis to classifing a case according to a year season. For example, results from December of 2017 are aggregated with the year of 2018, creating the year season of 2018, i.e., the year season of 2018 stated on December 1^st^ of 2017 and ended on November 30^th^ of 2018 (Fig 2).

### Receiving and processing data

Data in CSV format was retrieved from participating VDLs, and uploaded on SAS. A SAS script for each VDL was written to manipulate, clean, standardize, categorize, and format each of the original data to a common format having equal columns and pattern of information. A final standardized dataset for each participating VDL with PRRSV data was created using commands DATA STEP, PROC SQL, PROC SORT, and PROC MERGE on SAS. This method and the generation of final variables was accomplished through the following steps:

For the purpose of data cleaning, all received data was standardized through PROC SQL, PROC FORMAT, PROC SORT, and DATA steps on SAS. There was an effort to remove from the database cases linked to research (based on the field ‘reason for submission’), testing for pig exports (based on the field ‘reason for submission’), non-swine cases (based on the field ‘species’), cases having vaccine as specimen (based on the field ‘specimen’), and environmental samples (based on the field ‘specimen’).

At the end of the data processing step an aggregated dataset was formed by the merging data received from each of the VDLs into a consolidated dataset (Fig 1). The final aggregated dataset was stored at the SDRS database, located at ISU-VDL, with the historical data and transferred to the Power BI Desktop^®^ software (Microsoft Corporation, Redmond, WA) for data visualization. The accession ID`s were only used to gather, organize, and aggregate the dataset. Accession ID`s were completely removed from the aggregate dataset avoiding any possibility to link a result to an accession ID and/or to a participating VDL or its respective specific clients.

Results released by ISU-VDL and UMN-VDL during August to November of 2018 and standardized by snomed CT, and LOINC codes were messaged to a database server using the Health Level Seven International (HL7) format. The "Level Seven" refers to the seventh level of the International Organization for Standardization (ISO) seven-layer communications model for Open Systems Interconnection (OSI)–at the application level. Server database was accessed by an Open Database Connectivity (ODBC) interface provided by Microsoft^®^, and allowed the SAS application to access the data at the server using SQL password protected commands in a near real-time base. A final SAS algorithm using PROC SQL, PROC FORMAT, PROC SORT, PROC MERGE, and DATA steps processed the information using the standardized snomed CT, and LOINC codes, and consolidated the results with the historical aggregated data, and also processed data shared on a CSV format from SDSU-ADRDL, and KSU-VDL. In the end, this algorithm was capable of capturing the information from both sources, HL7 and CSV, to process and update the standardized database for the aggregated results.

#### Constructing dashboards, visualization charts, and online applications (APPs)

Final anonymized, aggregated, and standardized datasets were uploaded to Power BI^®^, and standardized charts for data visualization were structured. The first set of charts were composed by column bar charts representing the total number of cases and results over time. Percentage of positive results (PP) over the total cases were calculated and displayed in an area chart over time. Specimen type and age categories by year season were displayed in a 100% stacked column chart. The geographical distribution of positive results by PRRS type was plotted in a heat map. The charts were uploaded to an online dashboard using Power BI Pro^®^ (Microsoft Corporation, Redmond, WA) and organized in a PRRS application (APP) with the capability to produce dynamic dashboards with a set of predefined filters. The filters allow display of the information over time, by age category, specimen, tissue presence, and site state.

## Results

Historical anonymized data consolidated from all participating VDLs containing results for PRRSV were successfully aggregated between June of 2007 to November of 2018. Dynamic charts from the aggregated results were created in Power BI desktop^®^, which is available at www.fieldepi.org/SDRS under the PRRSV dashboard. Graphs with the number of cases or percentage of positive results were organized by year season and season, were successfully uploaded to an online dashboard in Power BI Pro^®^ and embedded in the website for visualization.

**Fig 3:**
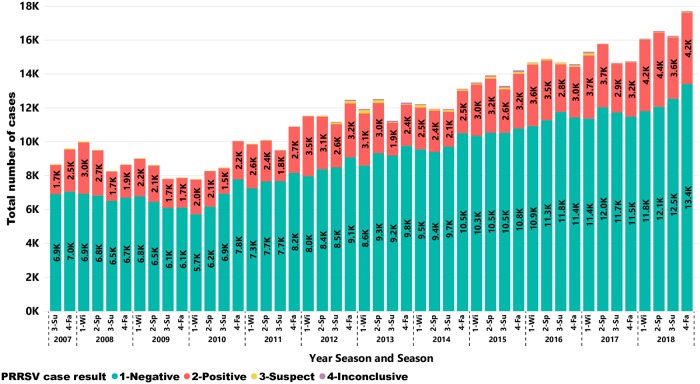
Results of PRRSV cases tested by RT-qPCR over time.

A total of 547,873 cases were tested by RT-qPCR for PRRSV in the period between June of 2007 through November 2018. Of the cases tested for PRRSV, 126,013 had a positive result. Fig 3 shows the number of cases tested over the years with the total number of cases increasing on average by 7.54% per year after 2009. The total number of cases tested for PRRSV almost doubled from 2009 to 2018 moving from an average of 8,339 to 16,641 cases per season.

Numbers are represented in 1,000 cases. Each bar represents a season within a year. Each color represents a case result, as indicated in the bottom section of the chart. Seasons are represented as follows: 1-Wi = Winter; 2-Sp = Spring; 3-Su = Summer, and 4-Fa = Fall.

**Fig 4:**
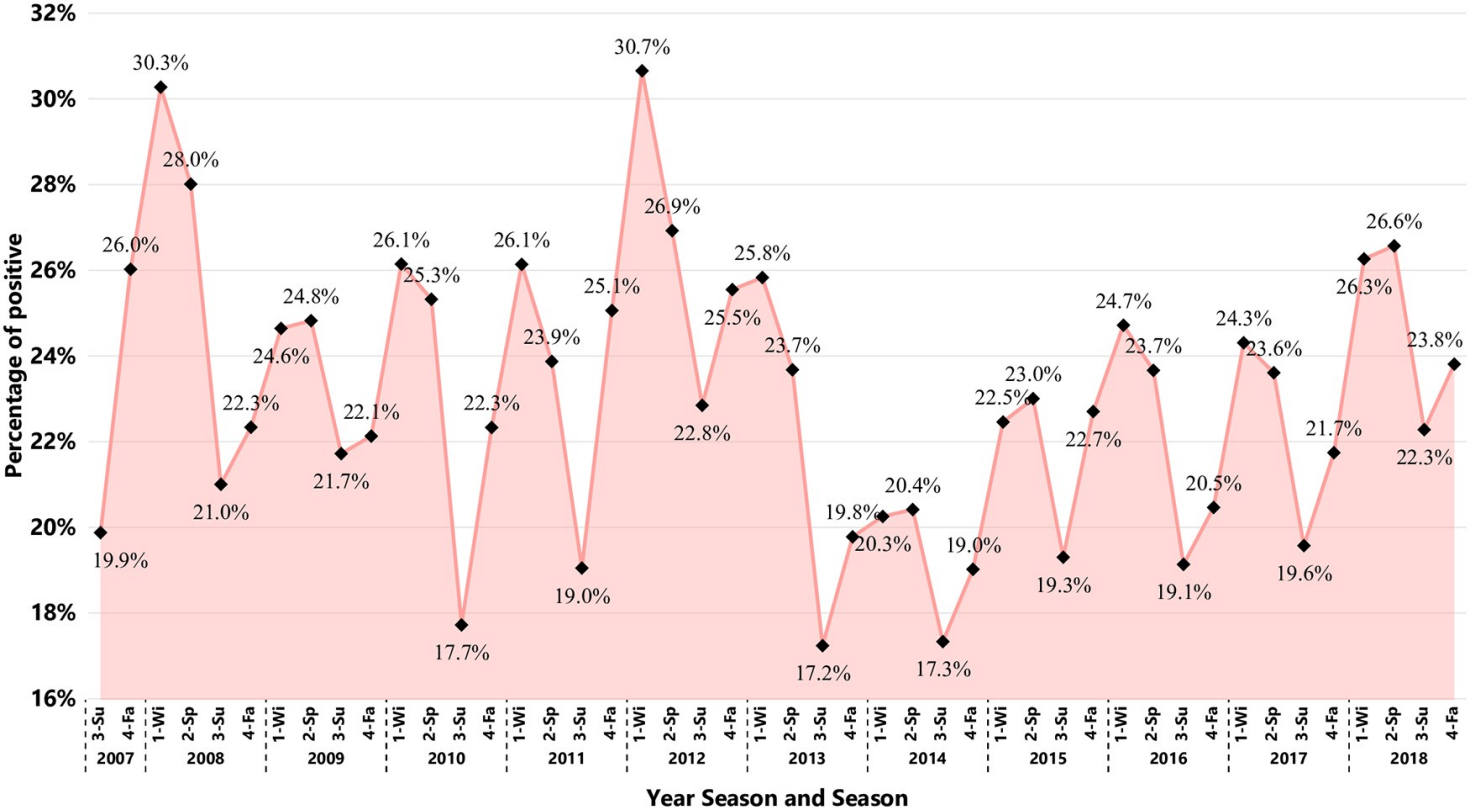
Percentage of RT-qPCR-positive results for PRRSV cases over the total number of cases per season. Each point represents a season within a year. Season are represented as follows: 1-Wi = Winter; 2-Sp = Spring; 3-Su = Summer, and 4-Fa = Fall.

As the number of cases tested for PRRSV increased over the years, there was also an increase in the number of positive cases. To better understand the pattern of positive results over time the percentage of positive cases from the total number of cases tested per season was calculated and plotted (Fig 4). There was an apparent seasonal trend in the detection of PRRSV with summer months having consistent smaller PP, and winter or spring months having the higher PP. The time period between Summer of 2013 and Fall of 2014 had the smallest PP cycle of PRRSV detection. After this period the PP started to slightly increase until the end of 2018.

**Fig 5:**
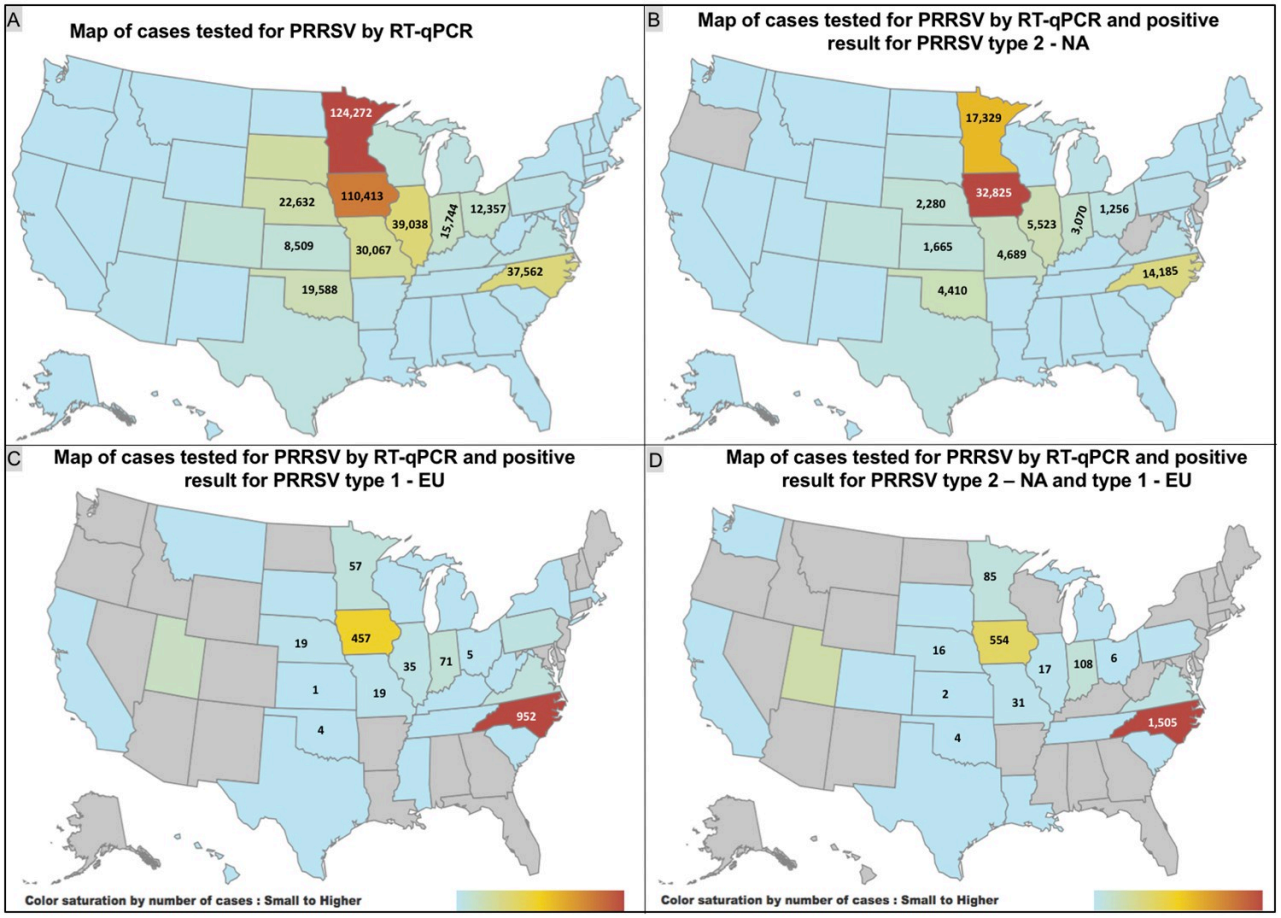
Geographical distribution of PRRSV RNA detection by RT-qPCR. **A**: overall distribution of all cases tested for PRRSV RNA. **B**: only cases with positive results for type 2 (North American)-PRRSV. **C**: only cases with for positive results type 1 (European)-PRRSV. **D**: only cases with a positive result for PRRS NA and positive result for PRRS EU. For VDL client confidentiality purposes, only states with hog inventory above 2 million heads [16] have numbers in the maps. Gray color shows the states with no cases.

The state geographical location of sample collection and the distribution of positive result for PRRS EU and PRRS NA RT-qPCR were compiled (Fig 5). States of Minnesota (MN) and Iowa (IA) were the 2 states with the highest number of cases tested for PRRSV. MN had the highest number of cases tested and overall PP of 21.01%. IA had a smaller number of cases submitted when compared with MN but the PP was 32.69%, which is 11.68 percentage points higher than MN. From the total number of 547,873 of cases tested for PRRSV, 126,013 had a positive result representing an overall PP of 23.00%. For 19,621 (15.57%) cases having a positive result, it was not possible to determine if it was PRRS EU or PRRS NA. From the remaining 106,392 positive cases, 94.76% (100,818) had a positive result for PRRS NA, 2.15% (2,289) had a positive result for PRRS EU, and 3.09% (3,285) had a positive result for PRRS NA and PRRS EU. IA had the predominant number of cases positive for PRRS NA (Fig 5B). On the other hand, PRRS EU was predominantly detected in samples from North Carolina (NC), followed by those from IA (Fig 5C). Cases having positive results for both PRRS types were predominantly from samples submitted from NC and IA (Fig 5D).

**Fig 6:**
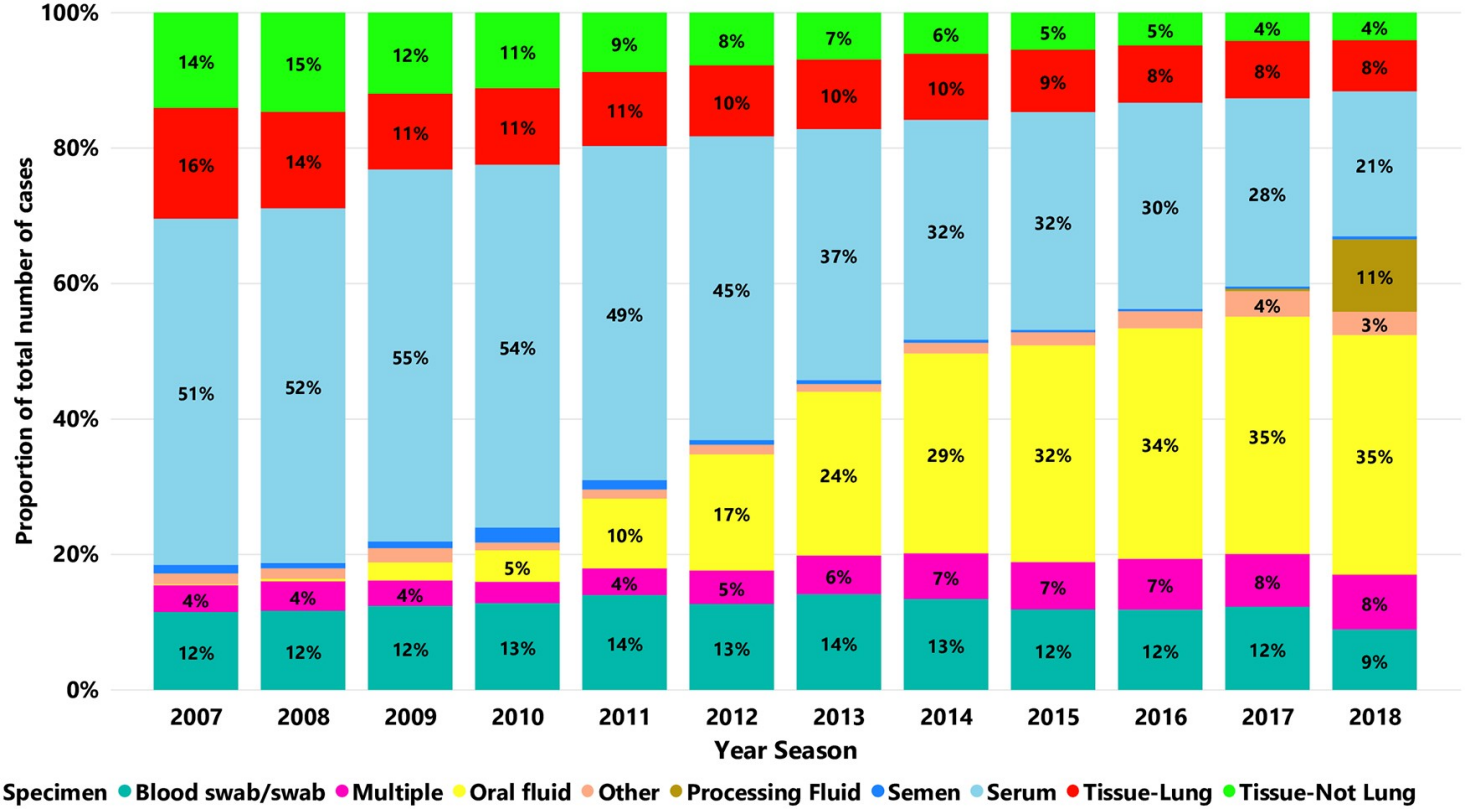
Proportion of specimens according to number of cases submitted for PRRSV RNA testing by RT-qPCR over time. Each bar represents a year season, and each color represents a specimen type.

Specimens tested for PRRSV over time are presented in Fig 6. From 2009 to 2018 there was a marked increase of oral fluid samples submitted for PRRSV testing. On average the submission using this specimen increased by 38.73% per year season after the calendar year of 2009, moving from 891 cases in 2009 to 23,532 cases in 2018. In 2018, processing fluid represented eleven percent of all cases tested for PRRSV with 7,095 cases tested. Conversely, there was an average reduction of 2.49% per year after the calendar year of 2009 in the proportion of submission using serum over the years, moving from 18,330 cases in 2009 to 14,239 cases in 2018. Number of submissions having specimen as Tissue-Lung, and/or Tissue-Not Lung samples remained practically stable over time, and increased only by 0.38% per year season after the calendar year of 2009, moving from 8,211 in 2009 to 8,533 cases in 2018. The proportion of cases with tissue (Tissue-Lung, and/or Tissue-Not Lung) decreased from 30.53% (11,131 of 36,451) of the total number of submissions in 2008 to 12.81% (8,533 of 66,567) in 2018.

**Fig 7:**
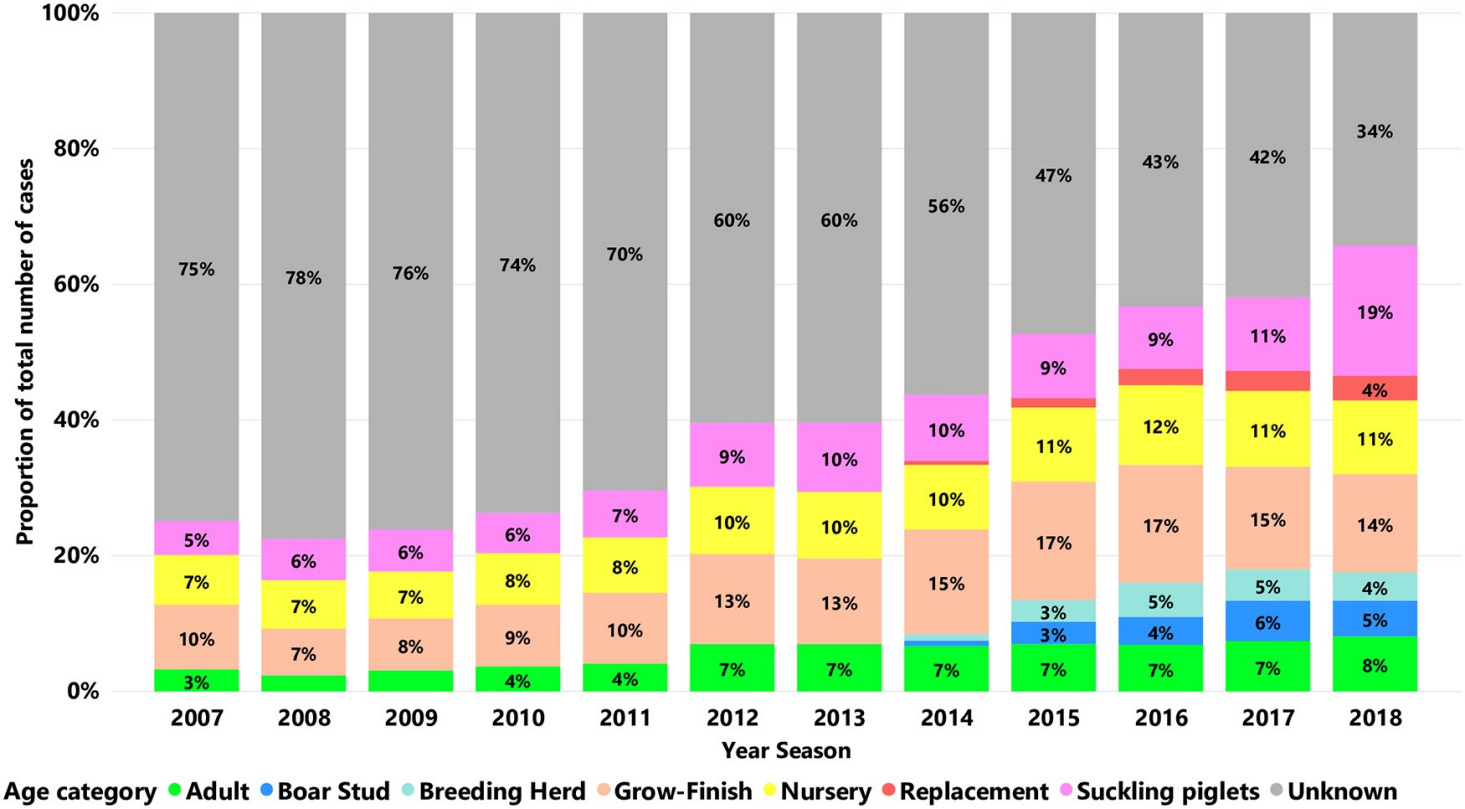
Proportion of age categories according to number of cases submitted for PRRSV RNA testing by RT-qPCR over time. Each bar represents a year season, and each color represents an age category.

The proportion of samples from each age category submitted for PRRSV testing by RT-qPCR is summarized in Fig 7. There was a marked improvement in the age category identification of cases submitted over time. More specifically, the age group ‘unknown’ decreased from 75% in 2007 to 34% in 2018.

## Discussion

This project demonstrates the capability and value of aggregating, consolidating, and summarizing anonymized data from multiple VDLs. More specifically, we were able to describe for the first time important macroepidemiological aspects associated with the detection of PRRSV RNA by RT-qPCR from the 4 major VDLs in the US (national level). Data from these 4 laboratories together represent 547,873 porcine cases tested in four NAHLN-accredited VDLs. PRRSV was chosen as a model pathogen because of its continuing global economic importance and control methods that rely heavily on biosecurity and surveillance. The methods described in this manuscript can be adapted and implemented to other pathogens affecting swine as well as to diseases of other species.

Total number of cases tested for PRRSV increased over time, doubling from 2009 to 2018. This increase was mainly observed in cases where tissue (specimen = Tissue-Lung or Tissue-Not Lung) was not present. Between 2007–2018, the specimens with increased number of submissions were blood swab/swab, which increased 30,26%, from 4,258 submissions in 2008 to 5,942 in 2018. Oral fluid increased from zero submissions in 2007 to 23,532 in 2018, and processing fluid increased from zero submissions in 2016 to 7,095 in 2018. In contrast, serum was the specimen with a decreased number of submissions (decreased 28.73% from 19,102 submissions in 2008 to 16,800 in 2018). These specimens are routinely used by the swine industry for monitoring purposes. A consistent increase in the number of cases was observed since 2011, which was when a general guideline to classify sow herds according to PRRSV status was published [17]. We speculate that producers and veterinarians rapidly adopted these sampling procedures to determine the PRRSV status of a herd, which has contributed to the increase in the number of submissions for monitoring purposes. The cases containing tissue samples had a decreased proportion of submissions over time. The relative increase of cases with “no tissue presence” suggests that the additional number of testing was done for monitoring and surveillance purposes. According to the MSHMP project, there was a substantial increase in the proportion of sow herds using modified live virus PRRSV vaccine after 2012 [8]. The increase in vaccine use potentially required more PRRSV testing to closely monitor and differentiate between vaccine-like virus and wild-type virus.

As previously discussed, most of the increased caseload over time was of cases with population-based specimens (oral fluids, processing fluids), and a relative reduction of cases having individual specimens such as serum, and swabs. In 2008 the detection of PRRSV in oral fluid was described [18] and guidelines for the collection and validation of this sample to monitor herds to PRRSV on a population-based standpoint was also described [19–21]. Validation of oral fluids-based monitoring protocols, combined with the prompt availability of RT-qPCR tests at the VDLs in 2010 [22], contributed to the increasing use of that specimen over time. In 2017 a novel specimen described as processing fluid was validated for PRRSV monitoring [14, 23, 24], and in 2018 this specimen represented almost 11% of all cases tested for PRRSV (56% of cases from sucking piglets). Oral fluid and processing fluid share some particular similarities. First, they can be applied to monitor PRRSV at a population level, processing fluid being applicable to younger piglets and oral fluid for older ages. Second, the number of samples required, the required labor, and the time commitment necessary for population-based sampling has been shown to be less than that of individual sample-based testing for pathogen surveillance purposes [19]. Those factors may have contributed to the increased adoption of oral fluids and processing fluids by the swine industry for PRRSV testing.

The percentage of cases testing positive (PP) for PRRSV has an apparently seasonal pattern of detection. For the period summarized, all summer seasons had consistently smaller PP when compared to the winter or fall months of the same year. The higher detection of PRRSV during winter months can be associated with the higher likelihood of mechanical transmission occurrence of the PRRSV from one farm to another during cold weather [25] when compared with warm weather [26]. The increase in the PP from summer to fall and winter months is commonly referred by the US swine industry as the “PRRS season”. The smallest PP window of time was detected during summer/2013 to fall/2014. During this period the US swine herd was responding to the transboundary introduction and epidemic of Porcine Epidemic Diarrhea Virus (PEDV) [27] and Porcine Deltacoronavirus (PDCoV) [28, 29]. Improvements on biosecurity measures, management practices, better cleaning and disinfecting measures for pig movement implemented to control PEDV may have also helped to reduce transmission of PRRSV between swine production sites. In the period of 2014–2018, the number of cases tested for PRRS increased by 26.07% (49,210 to 66,567). There was a trend in increased PP over years moving from an overall 19.26% PP in 2014 to 24.71% PP in 2018. The highest PP was detected in winter of 2012, with 30.66%. These findings coincide with the a relatively high PRRSV activity year, which led to the development of the MSHMP project [30].

The state of MN had the highest number of tests for PRRSV, but had a PP of 21.01% and was not among the states with highest overall PP results. NC had the highest PP with 37.76%, followed by IA with 32.69%. The relatively higher PP in IA and NC may be explained in part by the fact that these are also the 2 states with largest hog inventory in the US [16]. PRRSV NA was the predominant genotype detected in the US accounting for 94.76% of the positive results. The highest number of detections for PRRSV NA occurred in samples coming from IA. PRRSV EU had only 2.15% of detection and had highest detection in NC. This finding demonstrates that PRRSV NA is geographically detected in almost all of the US states. There was no detection of PRRSV NA in the states Oregon (OR), New Jersey (NJ), Delaware (DE), and Rhode Island (RI). Whereas the PRRSV EU detection was restricted to a smaller number of states with no detection in 20 states: OR, DE, NJ, RI, Nevada, Idaho, Wyoming, North Dakota, Arizona, New Mexico, Arkansas, Alabama, Georgia, Florida, Maryland, Connecticut, Vermont, New Hampshire, Maine, and Alaska.

Considerable improvement to identify age category information for cases submitted for PRRSV testing had been made over time reducing the unknown information. Starting in 2014, there was a significant number of cases identified as replacement, boar stud, and/or breeding herd (Fig 7). We believe this is due to better capturing information from cases, and does not necessarily mean an absence of cases including those age categories before 2014. This improvement is in part explained by the improvement of submission forms provided by participating VDLs and doesn`t necessarily mean that these age categories did not have samples submitted for testing during previous years and seasons. Even though this was an example of improvement in informing and recording the age category information there is still the need to keep improving this information in submission forms by submitters. Some technological improvements by the VDLs to better capture, and keep track of information like age category and site state may also improve capturing this information.

The bio-informatics tool, Swine Disease Reporting System (SDRS), that was developed, allows ongoing cooperation between participating VDLs to provide updated information of PRRSV detection over time, geographical space, specimen, and age group. The anonymized information uploaded in this tool does not contain confidential information such as client, veterinarian, owner, producer, specific location, or any other specific identifier of an individual VDL client.

To the best of our knowledge this is the first time that aggregated VDLs data is used in a consolidated project to share findings in standardized format to stakeholders [31]. It will allow providing aggregated information of PRRSV detection from participating VDLs thereby supplying additional information for stakeholders to analyze the aggregated results, ask informed questions to better understand the geographical distribution of the agent at a national or state level, and for monitoring, research, prevention, and swine production purposes. US producers, veterinary diagnostic laboratories, and any other stakeholder having a result dataset can make their own benchmarking comparison with the aggregated findings.

The SDRS has a robust dataset covering different production phases and can potentially provide synergistic information to other ongoing projects. Further exploring of this tool can give additional information on the pattern PRRSV detection and occurrence across different categories, and regions. As an example, SDRS has PRRSV detection information at a state level, PRRS CAP programs [32] or MSHMP project has the deepest information at premise ID level. Identifying increased proportions of cases testing positive on PRRSV RT-qPCR in a state where disease control programs are ongoing may serve as an alert and raise concerns for preventive enforcement in biosecurity measures as an attempt to avoid further pathogen spread in the area.

Interpretation of VDL data for epidemiologic purposes should be with great caution. Information presented in the SDRS contains results from samples originated on large scale, small scale, show pigs, and back yard production systems, thus inferences from aggregated results from one state regarding linkage to an individual producer are not plausible. Also, samples submitted for analysis at the VDLs are routinely collected in the field for monitoring or clinical diagnostic purposes without having a random sampling design. Implying prevalence, incidence and trends from non-random sampling cannot be made with statistical confidence. Non-random collection of samples across regions, and the actual number of cases tested for a region, state or other parameter can and will lead to a biased interpretation. Because of this, it is not plausible to argue that data from this study can be used to represent or imply PRRSV incidence or prevalence.

Efforts from all members involved with the process of submitting samples for veterinary tests can improve the quality of the final information for specimens, and more specifically for age categories. VDLs also play a crucial role in collecting this information. Improvement of submitters providing relevant information and VDLs capturing and storing such information from submission form is needed to improve the quality of the overall information, specifically for age, specimen and category.

Diagnostic data standardization across VDLs with use of LOINC codes and SNOMED CT aligned with the use of standardized messaging system such as HL7-messaging will allow performing continuing aggregation of data at a real-time basis for PRRSV and other pathogens. For the first time in the US, aggregated results for PRRSV tested by RT-qPCR from different VDL are made available and has the capability for continuous improvement and development of new features.

## Conclusions

Macroepidemiological aspects of PRRSV RNA detection by major US VDLs over time were described in this work. There was an increased number of cases tested for PRRSV, mainly driven by population-based specimens (oral fluids, processing fluids), which are typically used for disease monitoring purposes. An apparent cyclic pattern of PRRSV detection over time was identified with an indication of a seasonal trend with the highest detection occurring during winter and spring months, and the lowest detection occurring during summer months. PRRSV is geographically spread across the US, and PRRSV type 2 (PRRS NA) is the predominant strain type detected. PRRS type 1 (PRRS EU) is geographically restricted when compared with PRRSV type 2. There are opportunities to continue to improve submission information such as pig age and farm type in the submission forms, and/or collection of this information by the VDLs LIMS. The platform developed to consolidate and summarize results for this study was called Swine Disease Reporting System (SDRS). It consists of a user-friendly tool that provides aggregated information regarding swine pathogen detection from four participating VDLs with the ability to be updated on a near real-time basis. This tool allows rapid visualization of PRRSV detection and comparisons between the aggregated datasets with each individual stakeholder information. The SDRS tool has the ability to inform the swine industry regarding macroepidemiological aspects associated with PRRSV detection at the national or state level.

## Availability of data and materials

The aggregated information by season is publicly available online at www.fieldepi.org/SDRS under the PRRSV dashboard. Monthly reports with the major findings of PRRSV macroepidemiological aspects are compiled and distributed through email by the SDRS project and are available at the Swine Health Information Center (SHIC) under the Domestic Disease Monitoring Reports https://www.swinehealth.org/domestic-disease-surveillance-reports/. Restrictions apply to access to additional data, and SOP procedure used to aggregate the information due to the client and VDLs confidentiality, and are not publicly available. SOP procedure can be made available upon reasonable request and approval by SDRS principal investigator (Dr. Daniel Linhares at linhares@iastate.edu), SDRS project coordinator (Dr. Giovani Trevisan at trevisan@iastate.edu), and VDLs directors (Dr. Rodger Main at main@iastate.edu, Dr. Jerry Torrison at torri001@umn.edu, Dr. Jane Christopher-Hennings at jane.hennings@sdstate.edu, and Dr. Jamie Henningson at heningsn@vet.k-state.edu. We also state that the authors did not have any special access privileges to the data that others would not have.
